# Retraction Note: High diversity of *Morchella* and a novel lineage of the Esculenta clade from the north Qinling Mountains revealed by GCPSR-based study

**DOI:** 10.1038/s41598-021-93655-1

**Published:** 2021-07-01

**Authors:** Phonepaserd Phanpadith, Zhongdong Yu, Tao Li

**Affiliations:** 1 College of Forestry, Northwest A&F University, Yangling, 712100 Shaanxi China; 2 Sichuan Province Forest and Grass Seedling Station, Chengdu, 610082 China

Retraction of: *Scientific Reports* 10.1038/s41598-019-56321-1, published online 27 December 2019

The Editors have retracted this Article at the request of the corresponding author.

Several errors were discovered in the data-sets used to construct the phylogenetic trees, including duplicate sequences, incorrect accession numbers, and the inadvertent inclusion of a bacterial sequence. In addition, the EF1-α and RPB2 sequences cited in Table 2 were poorly amplified and sequenced, reducing the reliability of their application in tree constructions. As a result, the phylogenetic trees reported in the paper cannot be replicated.

Zhongdong Yu agrees with this retraction. Phonepaserd Phanpadidth and Tao Li have not responded to correspondence regarding this retraction.

